# IL-36γ-armed oncolytic virus exerts superior efficacy through induction of potent adaptive antitumor immunity

**DOI:** 10.1007/s00262-021-02860-4

**Published:** 2021-02-04

**Authors:** Min Yang, Esther Giehl, Chao Feng, Mathilde Feist, Hongqi Chen, Enyong Dai, Zuqiang Liu, Congrong Ma, Roshni Ravindranathan, David L. Bartlett, Binfeng Lu, Zong Sheng Guo

**Affiliations:** 1 UPMC Hillman Cancer Center, Pittsburgh, PA USA; 2 Department of Immunology, University of Pittsburgh School of Medicine, Pittsburgh, PA USA; 3 Department of Surgery, University of Pittsburgh School of Medicine, Pittsburgh, PA USA; 4 Department of Visceral, Thoracic and Vascular Surgery, University Hospital Carl Gustav Carus, TU Dresden, 01307 Dresden, Germany; 5 Department of Surgery, CCM/CVK, Charité-Universitaetsmedizin Berlin, Berlin, Germany; 6 AHN-Cancer Institute, Pittsburgh, PA USA

**Keywords:** Oncolytic virus, IL-36*γ*, Effector memory T cells, CD4^+^ T cells, CD8^+^ T cells, Immunotherapy

## Abstract

In this study, we aimed to apply the cytokine IL-36*γ* to cancer immunotherapy by constructing new oncolytic vaccinia viruses (OV) expressing interleukin-36*γ* (IL-36*γ*-OVs), leveraging unique synergism between OV and IL-36*γ*’s ability to promote antitumor adaptive immunity and modulate tumor microenvironment (TME). IL-36*γ*-OV had dramatic therapeutic efficacies in multiple murine tumor models, frequently leading to complete cancer eradication in large fractions of mice. Mechanistically, IL-36-*γ*-armed OV induced infiltration of lymphocytes and dendritic cells, decreased myeloid-derived suppressor cells and M2-like tumor-associated macrophages, and T cell differentiation into effector cells. Further study showed that IL-36*γ*-OV increased the number of tumor antigen-specific CD4^+^ and CD8^+^ T cells and the therapeutic efficacy depended on both CD8^+^ and CD4^+^ T cells. These results demonstrate that these IL36*γ*-armed OVs exert potent therapeutic efficacy mainly though antitumor immunity and they may hold great potential to advance treatment in human cancer patients.

**Supplementary Information:**

The online version contains supplementary material available at(10.1007/s00262-021-02860-4)

## Background

Recently, immune checkpoint inhibitors (ICIs) have been approved to treat a variety of cancers, such as melanoma, non-small cell lung cancer, renal cell carcinoma, and MSI-high colorectal carcinoma, resulting in great advances in cancer immunotherapy [1]. However, only a minority of human patients can benefit from these immunotherapeutic regimens. One major reason is that a large portion of patients are either naturally resistant to or eventually develop resistance to ICI immunotherapy [1]. There is a pressing need for new strategies to improve the clinical responsiveness of cancer immunotherapy. As a hallmark of cancer, the tumor microenvironment (TME) is highly immunosuppressive and contributes chiefly to resistance to immunotherapy [2]. Among the many properties of the TME, the lack of immune-stimulating “alarmin” cytokines in the TME poses a major obstacle for ICI immunotherapy [3, 4]. Therefore, effective delivery of cytokines to the TME is a promising strategy for the improvement of tumor immunotherapy.

Interleukin-36*γ* (IL-36*γ*), formerly IL1F9, is a member of the IL-1 family [5]. IL-36 has been shown to be induced in lining tissue cells such as keratinocytes and bronchial epithelia, as well as tissue innate immune cells such as macrophages and dendritic cells (DCs), and is believed to function as an “alarmin” in damaged tissue [6, 7]. IL-36*γ* binds to IL-36R (IL-1Rrp2) and IL-1RAcP and is involved in the activation of various immune cells such as DCs, T-cells, and natural killer (NK) cells [8–10]. Regarding the potential application of IL-36*γ* to cancer therapy, we have shown that IL-36*γ* transforms the TME and promotes type 1 lymphocyte-mediated antitumor immunity [9]. We have also shown that IL-36*γ* cooperates with T-bet in therapeutic DC-mediated promotion of ectopic lymphoid organogenesis in the TME, which is associated with antitumor efficacy in DC-mediated cancer vaccines [11]. The success of IL-36 as a tumor therapeutic agent relies upon tumor delivery of this cytokine, which increases antitumoral activity and reduces toxicity.

Oncolytic virus (OV)-based therapy is a new and promising type of tumor immunotherapy [12, 13]. These multimodal antitumor agents induce oncolysis of the infected cancer cells and tumor-associated stromal cells, usually in a form of immunogenic cell death (ICD). ICD is thought to further stimulate adaptive antitumor immune responses [12]. We and others have previously developed several genetically engineered oncolytic vaccinia viruses (VVs) to achieve tumor selectivity [14]. These VVs include the first one with a single deletion of the viral gene *tk* (now called vv.TK-) [15], the second one with a double deletion of viral genes *tk* and *vgf* (called vv.DD) [16], and the third one with a triple deletion of viral genes *tk*, *spi-1* and *spi-2* (called vv.TD) [17]. Our previous studies have shown that oncolytic VVs with these three genetic backbones display relatively higher tumor selectivity. Phase I clinical trials have demonstrated the safety of vvDD, but only minimum efficacy in most human patients with advanced solid cancers other than melanoma [18, 19]. In contrast, better efficacy of Pexa-Vec was shown in a phase II trial for hepatocellular carcinoma [20]. Despite promising clinical progress, new ways to improve OV-based cancer therapy are urgently needed.

Numerous studies have shown that OV-elicited antitumor adaptive immune responses play an essential role in OV-mediated therapeutic efficacy [12, 13, 21]. Various strategies have been designed and tested to further boost the antitumoral immunity of OV. These have included engineering OV to express heat-shock proteins, cytokines, or costimulatory molecules to enhance tumor immunogenicity [22–24], and combining OV with other immunotherapy regimens [25–27]. Recently, a number of studies indicated that OVs can turn a cold tumor into hot and thus combination of OVs with immune checkpoint blockade led to superior therapeutic efficacy in tumor models and in human melanoma patients [26, 28]. The first cytokine-armed OV approved by the FDA is a genetically engineered oncolytic herpes simplex virus expressing granulocyte–macrophage colony-stimulating factor called talimogene laherparepvec (T-VEC) [29]. The clinical benefit of T-VEC is, however, limited in advanced melanoma patients [29]. Therefore, synergistically combining the antitumoral activities of both VV and cytokines remains a difficult task.

One major goal of VV-based immunotherapy is to optimize VV viral vectors for improving immunogenicity while preserving tumor cell-selectivity. Targeting IL-1 gene family is an attractive and under-explored strategy because many VV-encoded genes are involved in suppressing the function of IL-1 family members [14]. SPI-2 inhibits the proteolytic activity of IL-1*β* converting enzyme (ICE) (also known as caspase-1) [30], and deletion of SPI-2 has been used in our vvTD vector and such modification increases tumor targeting and reduces toxicity [17, 31]. IL-36*γ* is a member of IL-1 gene super family and IL-36*γ* is also known to induced IL-1 expression. Therefore, we decided to determine whether we can increase immunogenicity using IL-36*γ*, particularly in combination in SPI-2 mutation.

In the current study, we constructed oncolytic VVs expressing an active form of IL-36γ. Our study was designed to leverage both direct oncolytic and tumor selectivity of VV and the immune stimulatory effect of IL-36*γ* for the induction of antitumor activity. We then tested the antitumoral efficacy of IL-36*γ*-armed VV in several murine syngeneic tumor models. The underlying immune mechanisms were subsequently investigated. Our study aimed to establish the feasibility of tumor-specific delivery of IL-36*γ* by VV and elucidate the mechanism of antitumoral synergism between these two tumor immune therapeutics.

## Materials and methods

### Mammalian cell lines

Murine cancer cell lines, B16 melanoma, MC38 colon cancer, and panc02 pancreatic cancer have been used often in our previous studies. Other mammalian cell lines, HEK293, HeLa, HepG2, MDA-MB-468, and CV-1, were originally obtained from ATCC (Manassas, VA). All mammalian cells were grown in Dulbecco’s Modified Eagle’s Medium (DMEM) supplemented with 100 U/mL penicillin and 100 µg/mL streptomycin, 2 mM L-glutamine, and 10% fetal bovine serum (FBS) (Gemini Bio-Products, West Sacramento, CA) in an incubator at 37 °C with 5% CO2.

### Generation of oncolytic VVs expressing recombinant IL-36*γ*

The plasmid (pcDEF-CD8SP-IL-36γ) contains a hybrid gene encoding the mature peptide (G13-S164) sequence of murine IL-36*γ* preceded by the human CD8*α* signal peptide sequence (as BamHI-EcoRI fragment) [9]. The DNA fragment was PCR amplified from this plasmid and cloned into pCMS1 [32]. This results in the new shuttle vector pCMS-IL-36*γ*. The insert was confirmed by DNA sequencing. To create new recombinant VVs in the genetic backbone for deletions of single, double, and triple viral genes, we infected CV-1 cells in six-well plates with wild-type (WR strain), vSC20, and vSP viruses at MOI of 0.1 for 2 h, respectively, and then transfected with the plasmid pCMS-IL-36*γ*. Two to three days later, the new virus was selected based on expression of YFP using multiple rounds of flow sorting and plaque purification using CV-1 cells, as described previously [32]. Through these procedures, we have made three novel oncolytic VVs with different VV backbones: vvTK-IL-36*γ* (*tk*-), vvDD-IL-36*γ* (*tk*-/*vgf*-), and vvTD-IL-36*γ* (*tk*-, *spi-1*- and *spi-2*-). These three backbone VVs are vvTK- (formerly vJS6), vvDD, and vvTD (formerly vSPT) for mutations of viral genes for TK only; both TK and VGF; or triple TK, SPI-1, and SPI-2.

### Viral replication in vitro

In vitro viral replication assays comparing IL-36*γ*-expressing OVs versus the parental ones were performed as described. Briefly, 1.0e5 MC38-luc or other cancer cells/well were plated on six-well plates and incubated overnight. The cancer cells were then infected with oncolytic VV at MOIs of 0.1 and 1.0 in 1.0 mL of 2% FBS-containing DMEM for 2 h. The infected cells were harvested after 24, 36, 48, and 72 h. Following harvest, the cell pellets were homogenized to release intracellular virions using Precellys® 24 Tissue Homogenizer (Bertin Instruments, Rockville, MD). The viral load of cell lysates was then determined by viral plaque assay in CV-1 cells.

### Oncolysis of cancer cells in vitro

Cancer cells (MC38, HepG2, MDA-MB-468) were plated at 1.0e4 cells/well in 96-well plates overnight and then infected with OV at MOI of 1.0. The number of viable cells was quantified with MTS assays using a kit according to the instruction of the manufacturer (Promega, Madison, WI). We plated 2.5e5 MC38 colon cancer per well in six-well plates, and the next day, we infected them with OVs at MOI of 0.5. At specified time points (24, 48, and 72 h), cells in the wells were harvested and viable cells were counted in the presence of trypan blue.

### Mice and murine tumor models and treatments

Female five- to six-week-old C57BL/6 J mice (B6; H-2 Kb) were purchased from the Jackson Laboratory (Bar Harbor, ME). They were housed in specific pathogen-free conditions at the University’s animal facility. For peritoneal carcinomatosis models, B6 mice were injected i.p. with 5.0e5 MC38-luc or 1.0e6 panc02-luc cancer cells, and five days later (or as indicated), mice were monitored for tumor growth via in vivo bioluminescence imaging using the Xenogen IVIS Optical In Vivo Imaging System (Caliper Life Sciences, Hopkinton, MA). Then mice were randomly divided into groups for treatments and injected i.p. with 200 μL PBS or oncolytic VVs at 1.0e8 pfu/200 μL unless indicated otherwise. Tumor growth was monitored periodically by imaging, and health of mice was monitored at least twice a week.

For characterization of infiltrated immune cells, a subcutaneous MC38 tumor model was established by injecting 5.0e5 MC38 tumor cells into the right flanks of B6 mice. When tumor size reached ~ 5 mm in dia, 1.0e8 pfu of the OV or PBS was injected intratumorally. Ten days later, tumor tissues were harvested and processed for immunochemistry for markers of CD3, CD4, and CD8 and DAPI staining.

In an additional experiment for depletion of certain types of immune cells, rat-anti-mouse monoclonal antibodies (Ab) were used to selectively deplete certain types of immune cells at indicated time points in the following manner: anti-mouse NK1.1 at 300 µg/injection [clone PK136, BioXCell, West Lebanon, NH], anti-mouse CD8 Ab at 250 μg/injection (clone 53–6.7, BioXCell), and anti-mouse CD4 Ab at 150 μg/injection (clone GK1.5, BioXCell) for depletion of NK, CD8^+^ T cells, and CD4^+^ T cells, respectively.

Long-term surviving mice bearing intra-peritoneal MC38 tumor treated with OVs were used for tumor cell re-challenge (~ 140 days after initial tumor cell inoculation). In those cured mice and naïve mice (control), 5.0e5 MC38-luc cancer cells were injected subcutaneously into the right flank, and 5.0e5 Lewis lung cancer cells into the left frank. Tumor appearance was recorded up to day 40 post re-challenge with cancer cells.

Tumor growth was monitored via digital caliper volume measurement and compared to naïve C57BL/6 mice inoculated with MC38-luc tumor implants at the same time. Tumor volume was calculated as: V_tumor_ (mm^3^) = (L × W^2^)/2.

### Flow cytometry and antibodies

BUV395 conjugated anti-mouse CD45 (clone: 30-F11), BUV737 conjugated anti-mouse CD4 (clone: GK1.5), Pacific Blue conjugated anti-mouse CD8a (clone: 53–6.7), PE-CF594 conjugated anti-mouse Foxp3 (MF23), PE conjugated anti-mouse Tim-3 (clone: 5D12), and Alexa Fluor 647 conjugated anti-mouse CD206 (clone: MR5D3) were purchased from BD Bioscience. PE-Cy7 conjugated IFN-γ (clone: XMG1.2) and FITC conjugated CD11b (M1/70) were purchased from eBioscience. Pacific Blue conjugated anti-mouse MHC II (clone: M5/114.15.2), PE conjugated anti-mouse Gr-1 (clone: RB6-8C5), BV510 conjugated anti-mouse CD24 (clone: M1/69), and APC-Cy7 conjugated anti-mouse F4/80 (clone: BM8) were purchased from Biolegend.

At indicated time points, lavaged cells were collected and analyzed using flow cytometry as previously described [26]. For IFN-*γ* staining, cells were stimulated for four hours with 50 ng/ml phorbol 12-myristate 13-acetate (PMA, Sigma) and 1 µg/ml ionomycin (Sigma) in the presence of 10 µg/ml Brefeldin A. After stimulation, cells were stained for antibodies to surface markers, followed by fixation permeabilization with Fixation and Permeabilization buffer (eBioscience) according to the manufacturer’s instructions. Then cells were stained with antibodies to intracellular markers. All the samples were applied to LSRII or Fortessa FACS (BD Biosciences) and analyzed by using Flowjo software (Tree star).

### Live animal imaging

MC38 colon cancer and panc02 pancreatic cancer were previously transduced with a lentivirus expressing firefly luciferase (MC38-luc and panc02-luc), thus allowing bioluminescence imaging. The growth of transplanted cancers was monitored by in vivo bioluminescent live animal imaging with the Xenogen IVIS 200 Optical In Vivo Imaging System (Caliper Life Sciences, Hopkinton, MA). Live animal bioluminescence imaging was performed for two purposes. One was to ensure that tumor implants were present and that groups had comparable tumor burdens, and the other was to monitor tumor progression, and was thus performed periodically after treatments.

### Assessment of animal health and survival

Animal health status and survival was monitored closely. Abdominal girth of mice bearing intraperitoneal tumor implants was monitored with caliper measurement and mice were sacrificed when girth exceeded 1.5 × original measurements. Mice either succumbed to their disease or were sacrificed when abdominal girth exceeded allowable measurements as described above. Mice with subcutaneous tumors were sacrificed when tumors reached a maximum diameter of 2 cm, became ulcerated, and/or interfered with murine activity.

### Immunofluorescence staining

Resected tumors were fixed for 2 h in 2% paraformaldehyde and incubated in 30% sucrose overnight. Sections were cut (5 µm) and stained with combined primary antibodies CD3 Alexa 488 (100,212, Biolegend), CD4 Alexa 594 (100,446, Biolegend), and CD8 Alexa 647 (100,727, Biolegend) and nuclei were labeled with Hoechst dye (bis benzimide, Sigma B-2283; 1 mg/100 ml in dH20). Images were acquired digitally from nine fields under each condition. Density of positive cells was evaluated by automated image analysis using Nikon Elements (Nikon Instruments Inc, Melville, NY). Percentage of CD3^+^ T cells, CD3^+^CD4^+^, and CD3^+^CD8^+^ T cells per area was calculated using the number of cells positive for the antibody versus the total number of cells. Student’s t-test was used to analyze statistical significance.

### Tumor microenvironment analysis

Once subcutaneous tumors reached 5 mm in dia, mice were treated intravenously with 1.0e8 pfu of OVs or PBS administered via tail vein injection. Tumor tissues were recovered two, four, and six days after viral or mock treatment and then homogenized using Precellys® 24 Tissue Homogenizer (Bertin Instruments, Rockville, MD). Single cells were collected for various assays.

### RT-qPCR

RNA was isolated from tumor homogenates of subcutaneous MC38-luc tumor implants using the RNeasy kit (Qiagen, Germantown, MD). The synthesis of cDNA was then performed using from 2 μg of RNA using qScript™ cDNA SuperMix (Quanta Biosciences, Inc., Gaithersburg, MD) and Dyad® Peltier Thermal Cycler (Bio-Rad, Hercules, CA). Quantitative PCR was then performed using TaqMan analysis with PerfeCTa® qPCR SuperMix (Quanta Biosciences, Inc.) on the StepOnePlus System (Life Technologies, Grand Island, NY). All PCR primers were purchased from Thermo Fisher Scientific (Waltham, MA).

Relative gene expression was compared to a housekeeping gene, either hypoxanthine–guanine phosphoribosyltransferase (HPRT1) or glyceraldehyde 3-phosphate dehydrogenase (GAPDH), and then expressed as fold increase (2^−ΔCT^), where ΔCT = CT _(Target gene)_ − CT _(HPRT1 or GAPDH)_.

### IFN-γ ELISpot assays

Briefly, at day 7 or at an indicated time after i.p. inoculation of 5.0e5 of MC38-luc colon tumor cells, tumor-bearing mice were treated i.p. with 1.0e8 pfu of OVs or PBS. On the specific time as indicated, intraperitoneal lavage was performed, during which 5 mL of 2% FBS-containing PBS was injected into the peritoneal cavity using an 18-gauge needle, and then the cavity was gently agitated before the volume was aspirated and repeated up to two times. Lavage fluid was collected and strained over a 100 μM cell strainer, and red blood cells were lysed using ACK Lysing Buffer and then strained over a 40 μM cell strainer. The CD8^+^ T cell population from 2.0e7 cells in the lavage was then isolated using an α-mouse CD8 microbead isolation protocol (Miltenl Biotec, San Diego, CA). Once isolated, 2.0e4 CD8^+^ T cells were stimulated with 4,000-rad-irradiated MC38 cells or control cancer cells (at 2.0e4) in RPMI 1640 media supplemented with 10% FBS at 37 °C, 5% CO_2_ for 24 h. Following incubation, the plates were appropriately washed and then incubated with biotinylated α-mouse IFN-*γ* antibody (mAb R4-GA2-Biotin, Mabtech, Inc., Cincinnati, OH). The plates were developed using Vectastain Elite ABC and AEC Peroxidase substrate (SK-4200) kits according to vendor protocols (Vector Laboratories, Inc. Burlingame, CA). Finally, the plates were read and analyzed using an ImmunoSpot™ analyzer and software (Cellular Technology, Ltd., Shaker Heights, OH).

## Statistical analyses

GraphPad Prism version 7 (GraphPad Software, Inc., San Diego, CA) was used to analyze the experimental data. Analysis was performed using one-way ANOVA test and nonparametric Student’s *t* test. Animal survival was assessed using Kaplan–Meier survival curves and analyzed using log rank (Mantel-Cox) test. A *p* value of < 0.05 was considered statistically significant. Standardized symbols are used in the figures, as follows: * *p* < 0.05; ** *p* < 0.01; *** *p* < 0.001; **** *p* < 0.0001; and ns: not significant.

## Results

### Construction and in vitro characterization of oncolytic poxviruses expressing IL-36γ

**Fig. 1:**
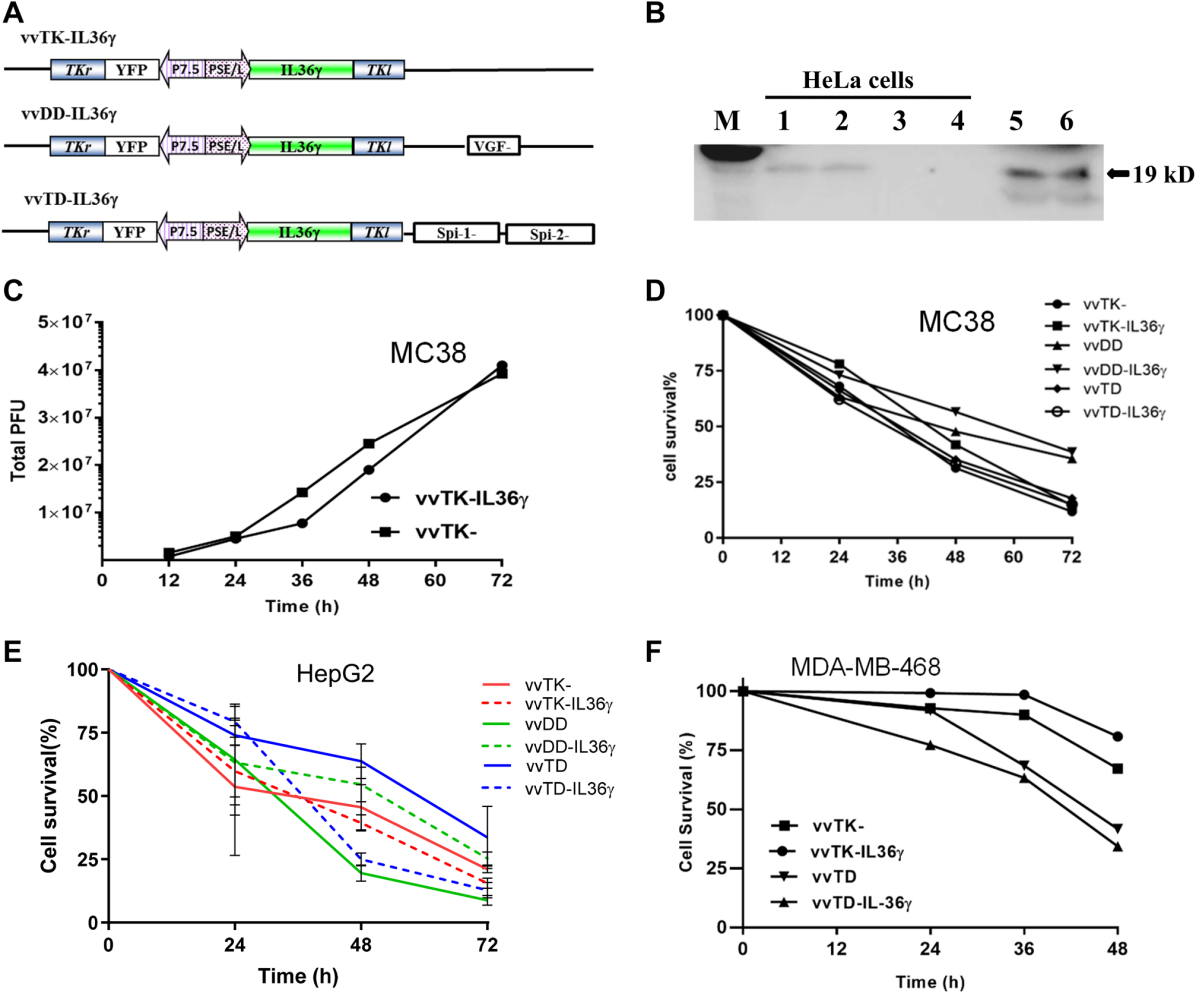
IL-36γ-armed VVs are OVs and produce the recombinant cytokine in infected cancer cells in vitro. **a** Three IL-36γ–armed oncolytic VVs containing various backbones with deletional mutations of viral genes. **b** Production and secretion of IL-36*γ* from infected HeLa cells. HeLa cells in six-well plate were mock-infected or infected with vvTK- or vvTK-IL-36*γ* at MOI of ~ 1.0. At 48 h post-infection, conditioned media were collected and subjected to western blot analysis. M: protein markers; lanes 1, 2: vvTK-IL-36*γ*; lane 3: vvTK-; lane 4: mock-infected. Lanes 5 & 6: B16-IL-36γ cells. **c** Viral replication in MC38 cancer cells. Harvested cells were lysed and the cell lysate was tittered using viral plaque assay. **d** MC38-luc cells were infected with OVs at MOI of 0.5 then harvested at varying time points. Cell suspensions were stained with 0.4% trypan blue solution and then viable cells were counted under visible light microscopy. **e** and **f** Oncolysis of virus-infected human cancer cells (HepG2 cells and MDA-MB-468 cells). Cancer cells in 96-well culture plates were infected with viruses at MOI of 1.0, then cell viability was assessed at 24, 36, 48, and 72 h after infection using MTS assays. These data are representatives of two or more independent experiments. For oncolysis in MDA-MB-468 cells, when comparing the two pairs of OVs (vvTK- and vvTL-IL36, versus vvTD and vvTD-IL36), *p* < 0.01. However, *p* > 0.05 when the 2 OVs in the same pair was compared

In order to determine whether we can increase antitumor efficacy of VV by IL-36*γ*, we constructed three IL-36*γ*-armed VVs, namely vvTK-IL-36*γ*, vvDD-IL-36*γ*, and vvTD-IL-36*γ*, by inserting an active form of IL-36*γ* into three VV backbones with different tumor selectivity and oncolytic activities (Fig. 1a). First, we verified the expression of IL-36*γ* from virus-infected HeLa cells. At 48 h post-infection with the control VV (vvTK-) and vvTK-IL-36*γ*, the secreted IL-36*γ* in the media was determined by Western blot analysis (Fig. 1b). We observed IL-36*γ* protein in the media from vvTK-IL-36*γ*–infected HeLa cells, but not from mock-infected, or control virus-infected HeLa cells. B16-IL-36*γ* melanoma cells, in which IL-36*γ*-expressing plasmid was stably transfected in our previous study [9], served as a positive control. These results demonstrated that the vvTK-IL-36*γ* virus-infected cancer cells synthesized and secreted IL-36*γ*.

We then compared replication efficiency and oncolytic activities of IL-36*γ*-expressing OVs with the parental OVs in three cancer cell lines. First, we infected MC38 colon cancer cells with vvTK- and vvTK-IL-36*γ*, and then examined their replication efficiency. Indeed, vvTK-IL-36*γ* and its parental virus vvTK replicated at similar rates (Fig. 1c). We then compared oncolytic activities among the three pairs of OVs in MC38 cancer cells (Fig. 1d) and HepG2 cells (Fig. 1e). We did not find difference between three pairs of OVs (*p* values: ns). Finally, we examined their oncolytic potency of two pairs of OVs in MDA-MB-468 human breast cancer cells. In this case, the vvTD pairs displayed slight better activity of oncolysis than vvTK- pair (*p* < 0.01) (Fig. 1f). Together, these data demonstrated that addition of the IL-36*γ* gene cassette to the VV genome did not reduce infectivity and oncolytic activities of OVs, and in some case, it might slightly enhance the oncolytic activity which is cell-dependent. The virus vvTD-IL-36*γ* displayed some better activity in most cancer cell lines; thus we have performed most in vivo experiment with this IL-36*γ*-armed OV and its parental virus vvTD as a control.

### IL-36*γ*-armed OVs induced stronger antitumoral activity in MC38 colon cancer model

**Fig. 2:**
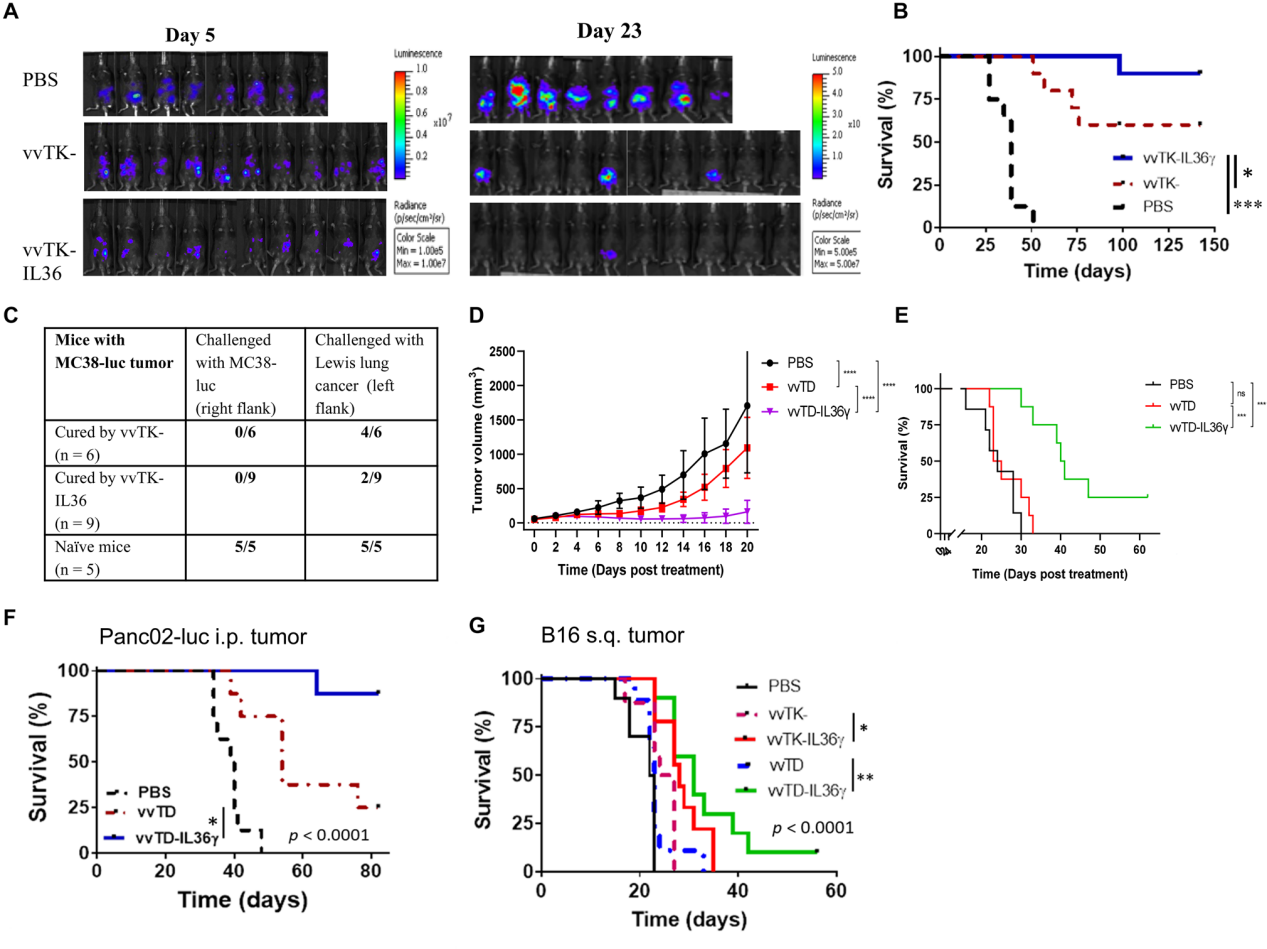
IL-36*γ*-armed OVs displayed potent antitumor effects in four murine tumor models. **Aa–c** B6 mice were inoculated i.p. with 5.0 × 10^5^ MC38-luc cells. On day 5, mice were imaged by bioluminescence to exclude mice with no tumor, and remaining mice were randomly divided into three groups and treated with PBS (*n* = 8), vvTK- (*n* = 10), or vvTK-IL-36*γ* (*n* = 10) at the dose of 1.0e8 pfu/mouse. **a** Tumor burden of mice on days 5 (the day of treatment) and 23 were determined via bioluminescence imaging. **b** Long term survival of MC38-luc tumor-bearing mice as Kaplan–Meier survival curves. *** *p* < 0.001 between PBS vs vvTK; *p* = 0.029 between vvTK vs vvTK-IL-36*γ* groups. **c** On day 140, the previously cured mice with vvTK- or vvTK-IL-36*γ*, along with a group of naïve mice, were re-challenged with cells of MC38-luc tumor (5.0e5 tumor cells) on the right flank and Lewis lung carcinoma (LLC, 5.0e5 cells) on the left flank. Tumor formation was observed twice a week until day 40. **d** Tumor curves of subcutaneous MC38-luc tumors treated with IL-36*γ*-OVs or PBS. **e** Long-term survival analysis of subcutaneous MC38-luc tumor-bearing mice after indicated treatments. Data were representative of two independent experiments. **f** Long-term survival of panc02-luc pancreatic tumor-bearing mice treated with indicated reagents was analyzed. *p* = 0.017, vvTD vs vvTD-IL-36γ; *p* ≤ 0.001 between PBS vs OV-treated groups. **g** Long-term survival of B16 melanoma-bearing mice treated with indicated reagents was analyzed. *p* = 0.024, vvTK- vs vvTK-IL-36*γ*; *p* = 0.002 vvTD vs vvTD-IL-36*γ*. Data were representative of two independent experiments. *n* = 6–10 for each group

We first examined the antitumoral activities of these OVs in an intraperitoneal MC38 tumor model as described previously [33]. On day 5 after tumor cell inoculation, mice were imaged and tumor-free mice were excluded. The remaining mice were then randomly divided into groups (Fig. 2a). Mice were subsequently treated with phosphate buffered saline (PBS), vvTK-, or vvTK-IL-36*γ* intraperitoneally (i.p.) at 1.0e8 pfu per mouse. They were monitored for toxicity and efficacy through appearance, tumor size, and survival. The parental virus vvTK- treatment prolonged survival of tumor-bearing mice (Fig. 2b). Treatment with vvTK-IL-36*γ* further prolonged survival (Fig. 2b) compared with the control vvTK-. Over the duration of the experiment (142 days), 6 mice (out of 10) treated with vvTK- were tumor-free, while 9 out of 10 mice treated with vvTK-IL-36γ were tumor-free (*p* = 0.029). To determine whether memory antitumor T cells were generated in the cured mice, we re-challenged the tumor-free mice with the same tumor cells (Fig. 2c). None of the cured mice grew any MC38 tumor. Interestingly, 7 out of 9 mice cured by vvTK-IL-36γ were protected from challenge with an unrelated Lewis lung cancer, whereas only 2 out of 6 mice cured by vvTK- were protected (*p* = 0.09; *t*-test). These results suggested that the adaptive antitumor immunity against MC38 tumor might have cross-reacted with Lewis lung cancer. As a control, both MC38 and Lewis lung cancer grew in 100% naïve mice. These results indicated that an IL-36*γ*-armed OV was a strong antitumoral agent with the ability to induce memory antitumor immune responses.

We also examined the kinetics of viral replication and IL-36γ expression in tumor tissues (Suppl. Fig. 1). IL-36γ was significantly increased in tumor homogenates on days 2 and 6 after administration of OVs as measured by ELISA (Suppl. Fig. 1a, d). The elevated levels of IL-36*γ* were also confirmed at the mRNA level using RT-qPCR (Suppl. Fig. 1b, e). mRNA of the viral marker gene A34R was detected at 60 h but reduced to basal levels by day 6 (Suppl. Fig. 1c,f). These data demonstrated that the viruses were replicated for a few days, and by day 6, they were reduced to low levels. In contrast, IL-36*γ* protein was sustained at a high level in the infected tumor tissues six days after OV administration.

### IL-36γ-armed OVs were efficacious in three other syngeneic tumor models

We also established subcutaneous MC38-luc and B16 models, and peritoneal Panc02-luc tumor models to test the activity of IL-36*γ*-armed OVs. In MC38-luc s.q. tumor model, vvTD inhibited tumor growth, and vvTD-IL-36*γ* showed improved antitumor efficacy (Fig. 2d). As a result, this translated into longer survival of animals (Fig. 2e). In the panc02 tumor model (Fig. 2f), vvTD-IL-36*γ* was also more effective than vvTD (*p* = 0.017) in prolonging mouse survival. In B16 tumor model (Fig. 2g), both vvTK-IL-36*γ* and vvTD-IL-36*γ* were more effective in prolonging survival than parental viruses vvTK (*p* = 0.024) and vvTD (*p* = 0.0016), respectively. These data collectively demonstrated that IL-36*γ*-armed OVs were potent antitumoral agents in multiple syngeneic murine tumor models.

### IL-36γ-expressing OV induced greater immune cell infiltration into the tumor

**Fig. 3:**
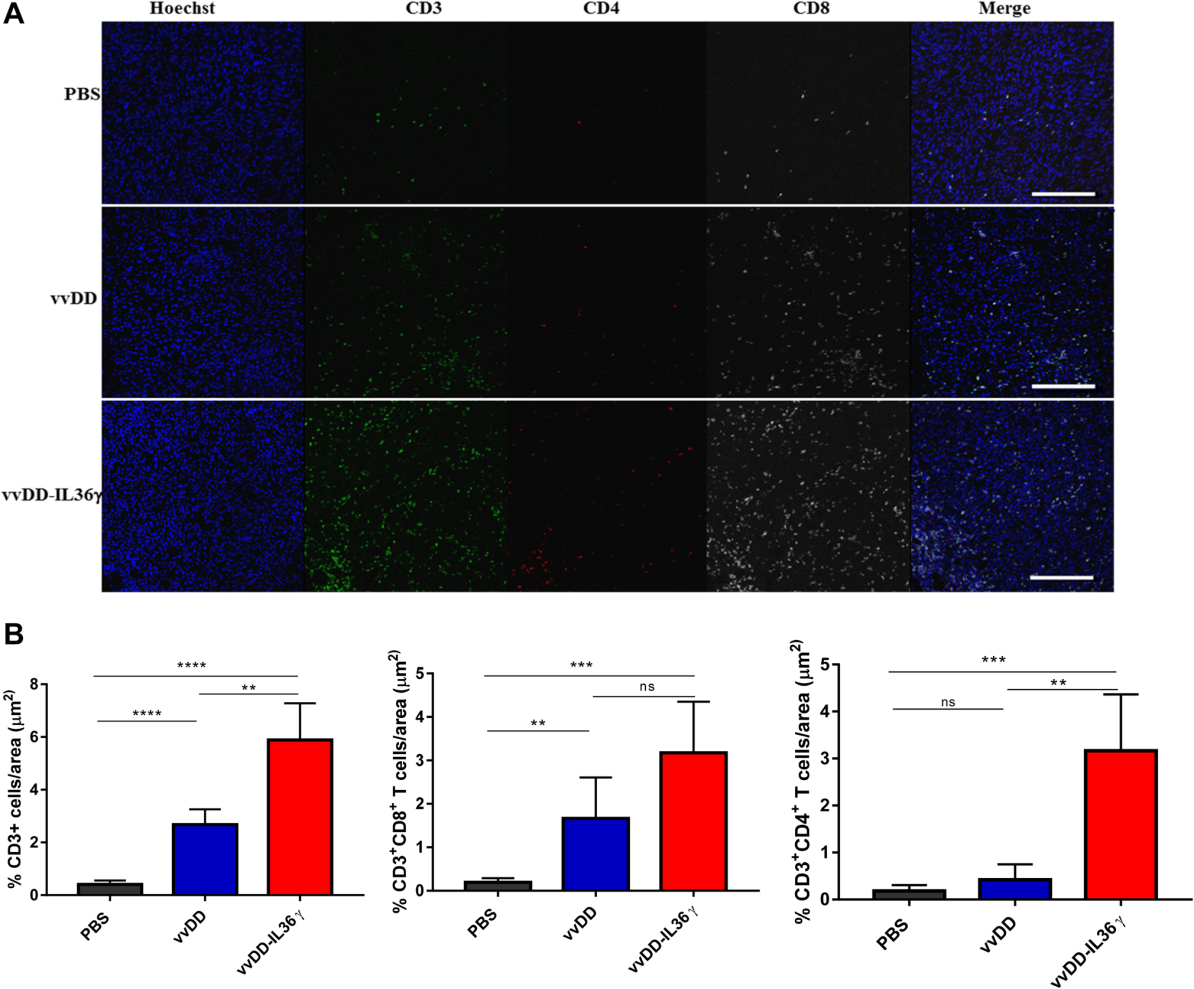
Treatment with IL-36*γ*-armed OV increased the number of T cells in MC38 solid colon tumor tissue. B6 mice were subcutaneously inoculated with 5.0e5 MC38 cancer cells. When the tumor size reached ~ 5 mm in diameter, PBS, vvDD, vvDD-IL-36*γ* (1.0e8 pfu per tumor) was injected intratumorally (*n* = 6 ~ 8/group). **a** Ten days post-treatment, tumor tissues were collected, fixed, and stained for CD3, CD4, CD8, and DAPI. Representative images from each group were presented. **b** Statistics of the percentages of CD3^+^ T cells, CD3^+^CD4^+^ T cells, and CD3^+^CD8^+^ T cells per area, ** *p* < 0.01, *** *p* < 0.001, **** *p* < 0.0001

We further studied the cellular mechanisms underlying stronger antitumoral activities of IL-36γ-OV using a MC38 subcutaneous tumor model. When tumors reached about 5.0 mm in dia, they were injected intratumorally with PBS, vvDD, or vvDD-IL-36*γ* at 1.0e8 pfu per mouse. On day 4 post-treatment, tumor tissue sections were analyzed using immunofluorescence microscopy for immune cell markers such as CD3, CD4, and CD8 for the assessment of the level of infiltrating T cells in the tumor tissues (Fig. 3a and b). vvDD increased the density of CD3^+^ cells in the tumor, and vvDD-IL-36*γ* increased it further. Additional analyses indicated that vvDD also increased CD8^+^ T cells, and vvDD-IL-36*γ* showed a trend of further increase of CD8^+^ T cells when compared to vvDD (Fig. 3a and b). In addition, vvDD-IL-36*γ* treatment resulted in more CD4^+^ T cell infiltration when compared to PBS or vvDD treatment (Fig. 3a and b). In summary, the IL-36*γ*-OV treatment induced higher levels of T cell infiltration into tumors.

### IL-36γ-armed OV-mediated therapy is dependent on CD4^+^ and CD8^+^ T lymphocytes

**Fig. 4:**
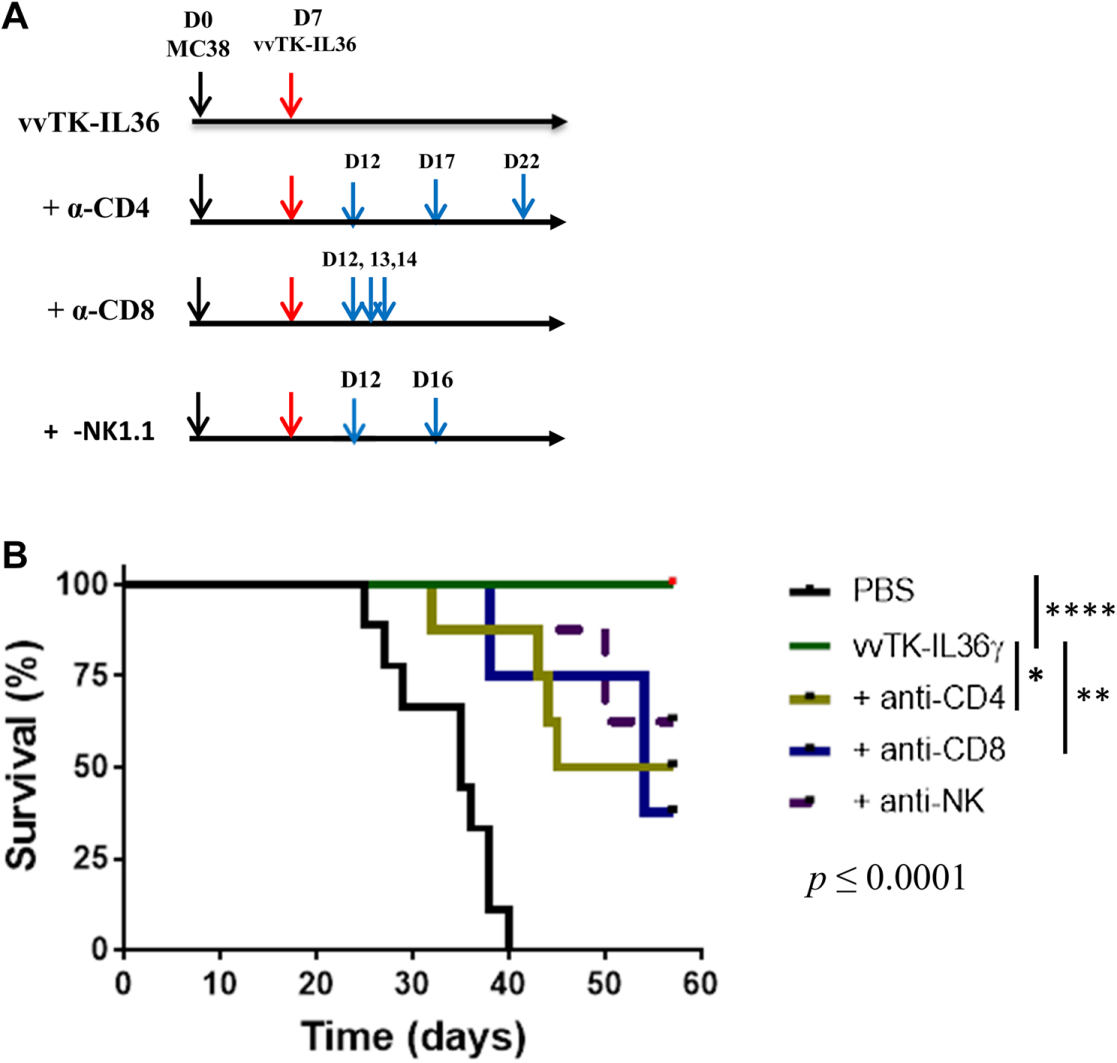
Therapeutic efficacy of IL-36*γ*-armed OV depends on multiple types of immune cells. Peritoneal MC38 tumor-bearing mice were imaged, randomized, and injected i.p. with PBS or 1.0e8 pfu of vvTK-IL-36*γ*. The mice treated with vvTK-IL-36*γ* were divided into four groups (*n* = 7–8) and further treated with PBS, anti-CD4 ab, anti-CD8 Abs, or anti-NK1.1 Abs as described in Methods. Mouse survival was monitored and Kaplan Meier analysis was performed. Statistical analyses: *p* = 0.025 for vvTK-IL-36γ versus vvTK-IL-36*γ* + anti-CD4; *p* < 0.01 for vvTK-IL-36*γ* versus vvTK-IL-36*γ* plus anti-CD8; *p* = 0.06 for vvTK-IL-36*γ* versus vvTK-IL-36*γ* plus anti-NK mAb treatment

Since T cells were increased by IL-36*γ*-armed OVs, we then investigated which types of lymphocytes were required for the therapeutic efficacy using the MC38 tumor model. On day 7 after tumor cell inoculation, MC38-tumor-bearing mice were imaged and randomly divided into five groups, with one group treated with PBS only and the other four groups treated with vvTK-IL-36*γ* (Fig. 4). The groups of vvTK-IL-36*γ*–treated mice were further treated with PBS, anti-CD4, anti-CD8, or anti-NK1.1 Ab, respectively, on a schedule as indicated (Fig. 4a). Mouse survival was monitored (Fig. 4b). Depletion of either CD4^+^ or CD8^+^ T cells significantly reduced vvTK-IL-36γ-mediated therapeutic efficacy (*p* < 0.01). We also found a trend—that the therapeutic effect was dependent on NK cells under these conditions (*p* = 0.06).

### IL-36γ-armed OV enhanced type 1 immune responses

**Fig. 5:**
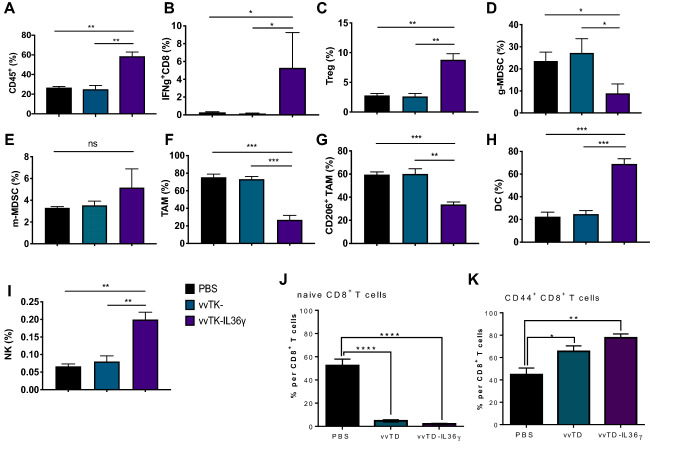
IL-36*γ*-armed OV promoted antitumor immunity via changing the TME. Mice were inoculated with 5.0e5 MC38-luc cells i. p., and seven days later, mice with similar sizes of tumor burden were randomly divided into three groups and treated with PBS or OVs (*n* = 6 for each group). Six days later, lavaged cells were analyzed using FACS. **a** Lymphocytes were gated based on the CD45 expressions in addition to Forward and Side scatters. Percentage of CD45^+^ lymphocytes out of total live cells. **b** Percentage of IFN*γ*^+^CD8^+^ T cells out of CD8^+^ T cells. **c** Percentage of Treg out of CD4^+^ T cells. **d** Percentage of CD11b^+^GR-1^hi^ g-MDSCs out of CD45^+^ population. **e** Percentage of CD11b^+^GR-1^int^ m-MDSCs out of CD45^+^ population. **f** Percentage of TAMs (CD24^−^F4/80^+^) out of CD11b^+^GR-1^−^MHCII^+^ population. **g** Percentage of CD206^+^ TAMs out of CD11b^+^GR-1^−^MHCII^+^CD24^−^F4/80^+^ population. **h** Percentage of DCs (CD24^+^F4/80^−^) out of CD11b^+^GR-1^−^MHCII^+^ population. **i** Percentage of Naïve CD8^+^ T cells gated as CD44^−^ CD62L^+^CD8^+^ out of total CD8^+^ T cells. **j** Effector memory-phenotype CD8^+^ T cells gated as CD44^+^ CD62L^−^CD8^+^ out of total CD8^+^ T cells. ** *p* < 0.01. *** *p* < 0.001. **** *p* < 0.0001

To gain further insight into the underlying mechanisms, we comprehensively studied immune cells lavaged from the abdominal cavity of intraperitoneally (*i.p.*) grown MC38-luc tumors. We collected cells lavaged from the peritoneal cavities of tumor-bearing mice on day 6 post-virotherapy and analyzed the immune cells using multi-color flow cytometry (Fig. 5 and Suppl. Fig. 2). We found that IL-36*γ*-OV induced an increase (in percentage) in total lymphocytes (Fig. 5a and Suppl. Fig. 2a). In addition, we showed that the frequency of IFN-*γ*^+^CD8^+^ T cells was greatly increased (Fig. 5b and Suppl. Fig. 2b). Interestingly, IL-36*γ* also increased the percentages of Treg cells, suggesting Treg cells might limit the antitumor efficacy of IL-36*γ* (Fig. 5c and Suppl. Fig. 2c). For the myeloid compartment, we found that the frequency of granulocytic myeloid-derived suppressor cells (g-MDSCs, especially CD11b^+^Gr-1^Hi^ subset), but not monocytic MDSCs (m-MDSCs) was reduced when treated with vvTK-IL-36*γ* compared to control OV and non-treatment (Fig. 5d, e and Suppl. Fig. 2d). In contrast, the percentage of total macrophages and M2-like tumor-associated macrophages (TAMs) (CD206^+^ TAM) were significantly reduced by IL-36γ-OV (Fig. 5f, g and Suppl. Fig. 2e, f; Suppl. Fig. 3a). The changes in M2 were also confirmed using another IL-36γ-OV (Suppl. Fig. 3a and c). In addition, the percentage of DCs was highly increased by IL-36*γ*-OV (Fig. 5h and Suppl. Fig. 2e). We also examined NK cells and found that IL-36γ-OV induced higher levels of NK cells compared to OV and PBS control (Fig. 5i, Suppl. Fig. 2g, and Suppl. Fig. 3b and d). Then we examined the activation status of CD8^+^ T cells in fractioned CD8^+^ T cell populations. In the mice treated with either OV, the population of naïve CD8^+^ T cells were reduced to less than 5% compared to ~ 55% in the PBS-treated mice (Fig. 5j). This happened concurrently with the increase of CD44^+^ CD8^+^ T cells, from ~ 45% in the PBS group, up to ~ 65% in vvTD-treated mice, and further up to 78% in mice treated with vvTD-IL-36*γ* (Fig. 5k). These results indicated that OVs promoted differentiation of naïve CD8^+^ T cells into memory and effector T cells (CD44^+^ CD8^+^) at the abdominal site, and IL-36*γ*-expression further enhanced this effect. Together, these data indicated that IL-36γ helped shape a more immunogenic TME and enhanced adaptive antitumoral immunity.

### IL-36γ-armed OV promoted tumor antigen-specific CD8^+^ T cells

**Fig. 6:**
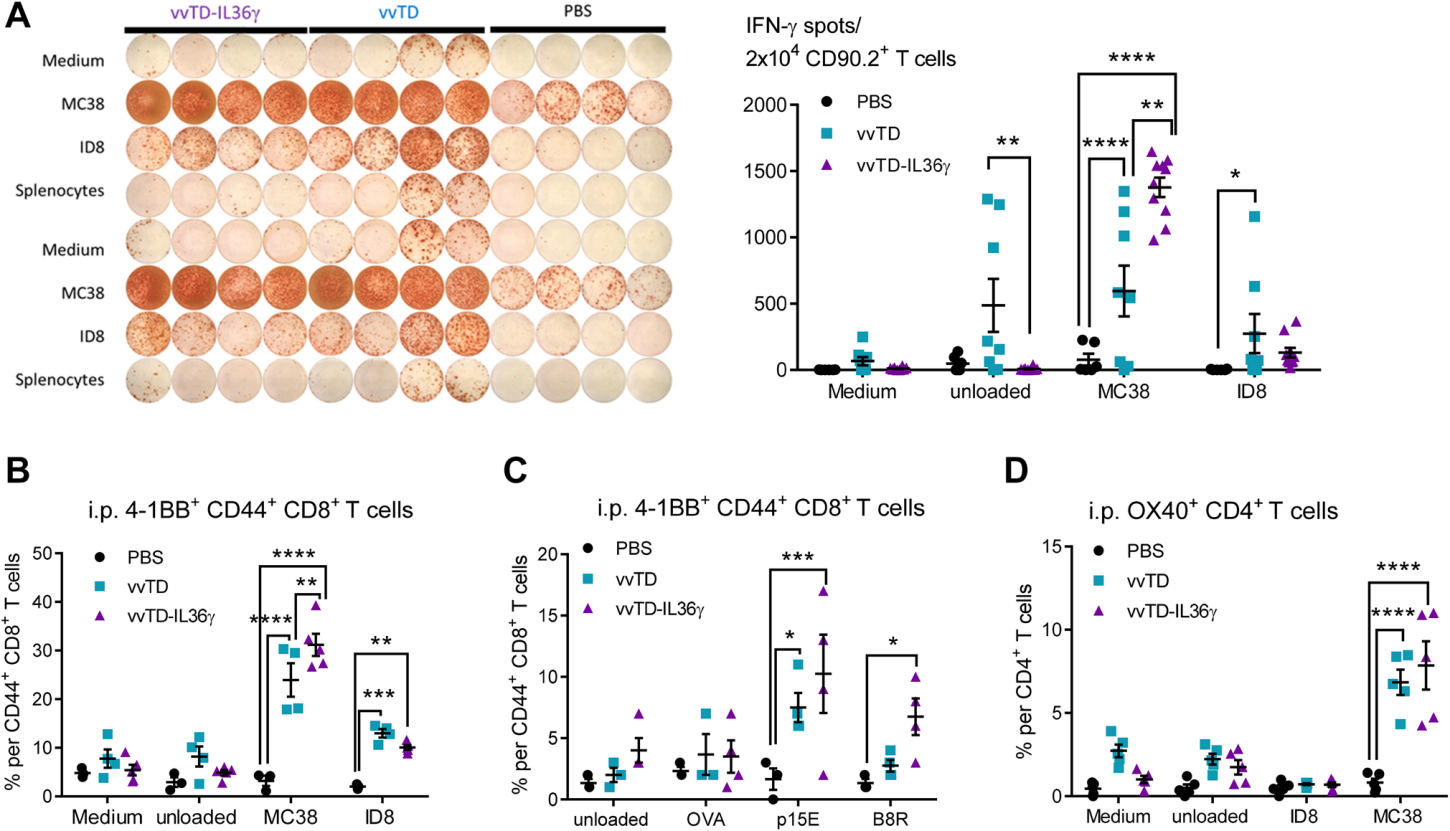
vvTD-IL-36*γ* enhanced recognition of murine MC38 colon adenocarcinoma cells by intraperitoneal 4-1BB^+^ CD8^+^ T cells on day 6 after oncolytic virotherapy. **a** Representative image of IFN*γ* ELISpot assay of 2.0e4 CD90.2^+^ T cells isolated from lavage specimens on day 6 post-oncolytic virotherapy and co-cultured 1:1 with specific (MC38) and unspecific (medium, ID8, splenocytes) target cells and analysis of ImmunoSpot™ counted spots. **b** 4-1BB^+^ CD44^+^ CD8^+^ T cells showed enhanced MC38-specific activation assessed by 4-1BB surface expression after co-culture assay with MC38 and unspecific target cells (medium, unloaded splenocytes, ID8 cells). **c** Percentage of 4-1BB^+^CD44^+^ CD8^+^ T cells, following co-culture assay with p15E_604–611_ and B8R_20–27_ loaded splenocytes and OVA_257–264_ and unloaded splenocytes as unspecific targets, revealed augmented 4-1BB dependent activation by retroviral peptide p15E_604–611_ and B8R_20–27_. **d** Percentage of OX40^+^ CD4^+^ T cells per total CD4^+^ T cells following in vitro co-culture assay with MC38 and unspecific targets as negative controls. ** *p* < 0.01; *** *p* < 0.001; **** *p* < 0.0001. The data were representatives of two or three independent experiments

We also examined the numbers of tumor antigen-specific T cells in the MC38 tumor-bearing mice treated with PBS, vvTD, or vvTD-IL-36*γ* on day 6 post-treatment (Fig. 6). An IFN-γ ELISpot assay was performed with T cells co-incubated with radiated tumor cells. Co-incubation with irradiated MC38 cancer cells led to ~ 520 spots per 2.0e4 T cells for vvTD and ~ 1300 spots for vvTD-IL-36*γ* (*p* < 0.01) (Fig. 6a). When co-incubated with irradiated ID8 ovarian cancer cells (control cells), there was a much lower number of spots, suggesting tumor cell specificity of the T cells. Similar results were obtained using T cells isolated from lavaged specimens from mice at day 11 after virotherapy (Suppl. Fig. 4a). Yet, in the spleen, there were few tumor-specific T cells in mice treated with vvTD-IL-36*γ* (Suppl. Fig. 4b). The reason is not clear, even though we could speculate that IL-36*γ* probably promoted tumor-specific T cell traffic to tumor tissue area.

During antitumor and antiviral adaptive immune response, naive antigen-specific CD8^+^ T cells undergo a highly orchestrated activation process [34, 35]. Previously, activation-induced expression of 4-1BB^+^ (or CD137^+^) has accurately correlated with naturally occurring tumor-reactive T cells in cancer patients [36]. Therefore, 4-1BB has been used to identify tumor or viral antigen-specific CD8^+^ T cells [37]. We also examined the frequency of 4-1BB^+^CD44^+^CD8^+^ T cells in the TME (Fig. 6b). Cell-surface receptor-CD44 was applied as a marker for antigen-experienced, effector and memory T cells [38]. We found that vvTD-IL-36*γ* induced more 4-1BB^+^ CD8^+^ T cells than vvTD (Fig. 6b) (*p* < 0.01 between vvTD vs vvTD-IL-36*γ*). Next, we examined both tumor-antigen- and viral antigen-specific CD8^+^ T cells (Fig. 6c). We had picked a well-studied tumor-specific self-antigen p15E, which is expressed by endogenous retrovirus in a variety of murine cancer cell lines and was previously defined to function as a tumor rejection antigen [39–41]. The isolated T cells were re-stimulated with a control peptide from OVA (OVA_257–264_), tumor antigen peptide from p15E (p15E_604–611_), and viral antigen peptide from B8R (B8R_20–27_). When unloaded or stimulated with OVA, 2–4% of T cells were 4-1BB^+^CD8^+^ effector T cells from mice treated with PBS, vvTD, or vvTD-IL-36*γ*. When re-stimulated with p15E peptide, 4-1BB^+^CD8^+^ T cells were up to 2% from mice treated with PBS, ~ 7.5% with vvTD, and up to 10.5% in vvTD-IL-36*γ* (Fig. 6c). Interestingly, we observed a similar but somewhat modest increase of viral antigen-specific 4-1BB^+^ T cells by vvTD-IL-36*γ*. (Fig. 6c). To compare these results with the activation of CD4^+^ T cells, we examined the surface expression of OX40 on CD4^+^ T cells and found an increased percentage of OX40^+^ CD4^+^ T cells in the vvTD and vvTD-IL-36*γ* treatment groups after overnight co-culture with MC38 cells in comparison with PBS (Fig. 6d).

In summary, these results strongly suggested that, compared to parental OV, IL-36*γ*-OV increased NK cells, DC, yet reduced g-MDSCs and M2-like TAMs, and enhanced number and activity of tumor-antigen-specific T cells and thus enhanced antitumoral efficacy.

## Discussion

In our previous study, we demonstrated that expression of IL-36*γ* in tumor cells greatly enhanced adaptive antitumor immunity [9]. In the current study, tumor tissue delivery of IL-36*γ* was achieved using genetically engineered IL-36*γ*-OV, making IL-36*γ* therapy more feasible in the clinical setting. Insertion of IL-36*γ*-expressing module also increased the antitumoral efficacy of OV. Our study has established a new combinatorial approach to leveraging the antitumoral activities of both IL-36*γ* and OV.

Previous studies with a variety of OVs indicated that this class of antitumor agents are promising, yet improvement in efficacy is badly needed. We and others have studied and improved oncolytic VVs over the last two decades. One of the best genetically engineered oncolytic VVs from our group has been the virus backbone called vvDD, in which the deletion of two viral genes encoding thymidine kinase and vaccinia growth factors enhanced its tumor selectivity without greatly diminishing its oncolytic potency [16]. However, two phase I clinical trials have shown its safety, but very limited efficacy in patients with advanced solid cancer [18, 19]. Thus, better VV viral backbones with higher baseline efficacy are needed. In addition, it is interesting that different types of cancers may have different susceptibility to different OVs; thus careful evaluation of a particular OV in that target cancer type is needed before clinical application of this OV in patients with that type of cancer. The differences in efficacy might be due to differences in infectivity, oncolysis, and immunogenicity of the tumor. This may also extend to which cytokine to be used for that type of cancer, as there are different TME for different cancers in which cytokine may function to a different degree. In this sense, a lot of more work needs to be performed in the future.

VV has developed many immune evasion mechanisms [14, 42]. Genetic engineering to target these mechanisms can reduce toxicity, increase tumor specificity, and increase tumor immunogenicity. Therefore, one major goal of VV-based immunotherapy is to optimize VV viral vectors for improving immunogenicity. We have reviewed 20 virus-encoded genes whose products modulate innate and adaptive immunity. One of major immune evasion mechanisms of VV focuses on IL-1 family cytokines [43, 44]. IL-36*γ*, which is a member of the IL-1 gene family and has been shown to induce expression of IL-1, is considered mechanistically synergistic with VV in tumor immunotherapy. Indeed, our study has provided experimental proof supporting this notion.

Addition of IL-36*γ* improved OV immunotherapy in several ways based on our detailed immunological characterization of both the TME and systemic adaptive immune responses. First, we showed that, compared to control VV, IL-36*γ*-VV greatly enhanced tumor site adaptive immune responses by increasing the tumor infiltration of CD3^+^ T lymphocytes, including both CD4^+^ and CD8^+^ T cells. In addition, IL-36*γ* promoted qualitative changes such as increases in IFNγ production by CD8^+^ tumor-infiltrating lymphocytes and DCs and decreased M2 TAMs and MDSCs. We have observed that IL-36 reduced the CD11b^+^Gr-1^hi^ subset of g-MDSCs significantly. This particular subset could directly contribute to tumor growth and vascularization by producing MMP9 and differentiating into endothelial cells [45].

Cell depletion using antibodies against CD4, CD8, and NK1.1 in the MC38 tumor model showed that the therapeutic efficacy of this IL-36*γ*-armed OV depended on both CD4^+^ and CD8^+^ T cells, and partially on NK cells. To our knowledge, this is the first study to show that OV-elicited adaptive antitumor immunity is dependent on CD4^+^ T cells in addition to CD8^+^ T cells. In this context, Fonteneau and team have found that OVs sensitize human cancer cells for NY-ESO-1 tumor antigen recognition by CD4^+^ effector T cells [46]. In the future, we would like to explore the mechanisms by which OV stimulates antigen presentation via MHC class II and determine the nature of these CD4^+^ T cells.

Other analysis further revealed that IL-36*γ* not only promoted bulk T cell changes in the tumor environment, it also enhanced antigen-specific antitumoral immune responses. We have used p15E to represent tumor antigens for human and murine colorectal cancers [39–41, 47–49], and B8R, which is a dominant antigen epitope from VV [50]. When compared to control VV parental virus, IL-36*γ*-armed VV generated more tumor antigen-specific T cells. Interestingly, IL-36*γ* also increased the number of viral antigen-specific T cells. These results indicate that IL-36*γ* improves adaptive immunity not only against tumor cells, but also against the virus, further ensuring both antitumoral efficacy and safety against potential viral infection with the combined therapy. In fact, the antiviral immunity could potentiate the immunotherapeutic efficacy against cancer by OVs [51]. Our results warrant the clinical study of human IL-36γ-armed OV in patients with advanced solid cancers or blood cancers.

## Conclusions

IL-36*γ*-armed OVs provide unique synergism and promote antitumor adaptive immunity and modulate TME. IL-36*γ*-OV had dramatic therapeutic efficacies in multiple murine tumor models, leading to complete cancer eradication in large fractions of mice in some tumor models. The OV induced infiltration of lymphocytes and DCs and decreased MDSCs and M2 TAMs. Its therapeutic efficacy depended on not only CD8^+^ T cells, but also CD4^+^ T cells even in the late phase of therapy, shown for the first time for an OV in antibody-mediated cell depletion experiment. These data provide a solid foundation for clinical evaluations of IL-36*γ*-armed OV in human patients with solid tumors.

### Supplementary Information

 Supplementary file 1 (pptx 28,971 kb)

## Data Availability

All data associated with this study are available in the main text or the supplementary materials. Key materials are available upon request.
