# Supplementary material for: IL-36γ-armed oncolytic virus exerts superior efficacy through induction of potent adaptive antitumor immunity
Source: Cancer Immunol Immunother. 2021 Feb 4;70(9):2467–81. doi: 10.1007/s00262-021-02860-4 (PMC8360872; doi:10.1007/s00262-021-02860-4)
Supplement: Supplementary file 1 — (pptx 28,971 kb) [file 262_2021_2860_MOESM1_ESM.pptx]

## Slide 1
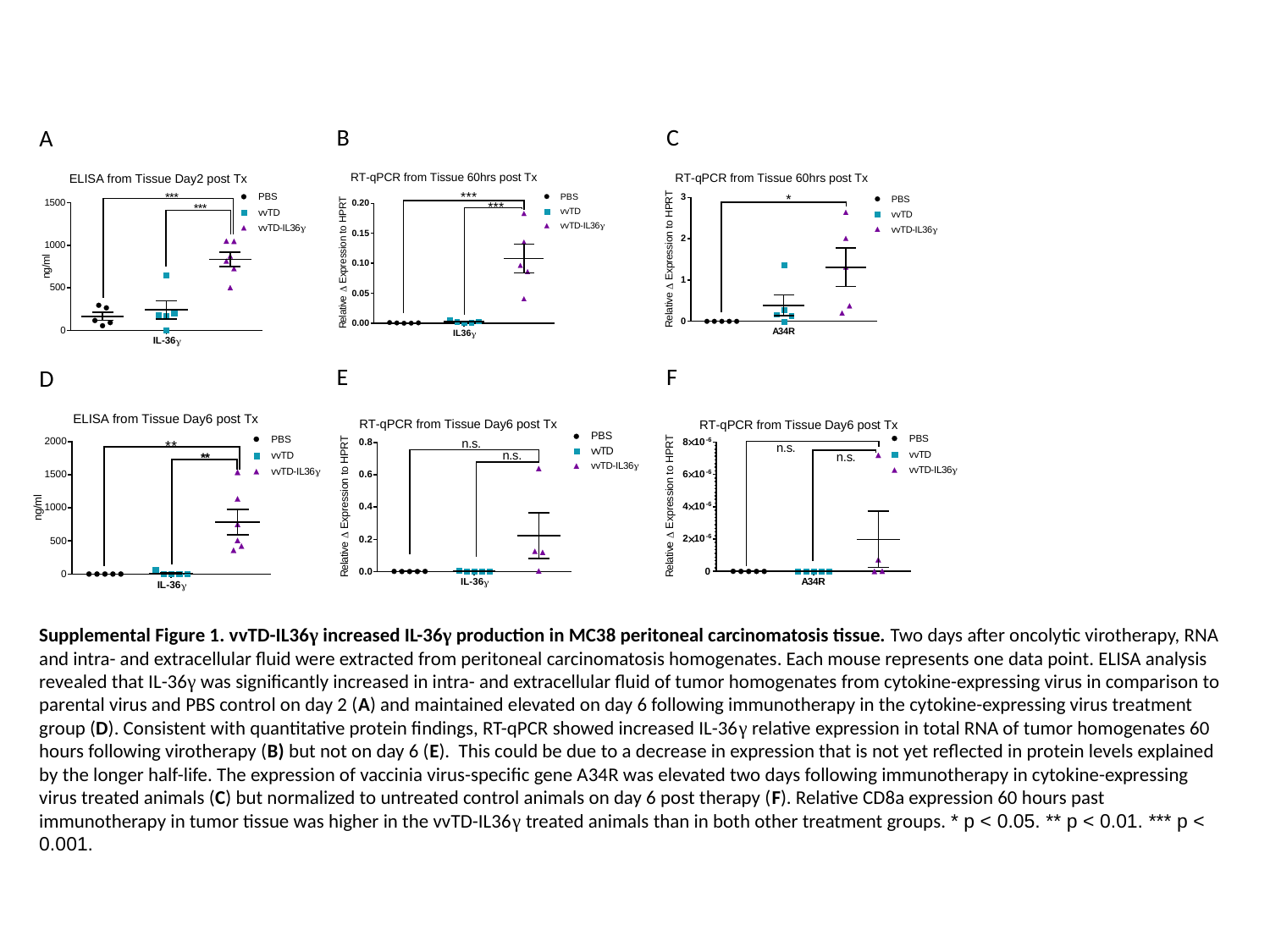

B
C
A
E
F
D
Supplemental Figure 1. vvTD-IL36γ increased IL-36γ production in MC38 peritoneal carcinomatosis tissue. Two days after oncolytic virotherapy, RNA and intra- and extracellular fluid were extracted from peritoneal carcinomatosis homogenates. Each mouse represents one data point. ELISA analysis revealed that IL-36γ was significantly increased in intra- and extracellular fluid of tumor homogenates from cytokine-expressing virus in comparison to parental virus and PBS control on day 2 (A) and maintained elevated on day 6 following immunotherapy in the cytokine-expressing virus treatment group (D). Consistent with quantitative protein findings, RT-qPCR showed increased IL-36γ relative expression in total RNA of tumor homogenates 60 hours following virotherapy (B) but not on day 6 (E). This could be due to a decrease in expression that is not yet reflected in protein levels explained by the longer half-life. The expression of vaccinia virus-specific gene A34R was elevated two days following immunotherapy in cytokine-expressing virus treated animals (C) but normalized to untreated control animals on day 6 post therapy (F). Relative CD8a expression 60 hours past immunotherapy in tumor tissue was higher in the vvTD-IL36γ treated animals than in both other treatment groups. * p < 0.05. ** p < 0.01. *** p < 0.001.

## Slide 2
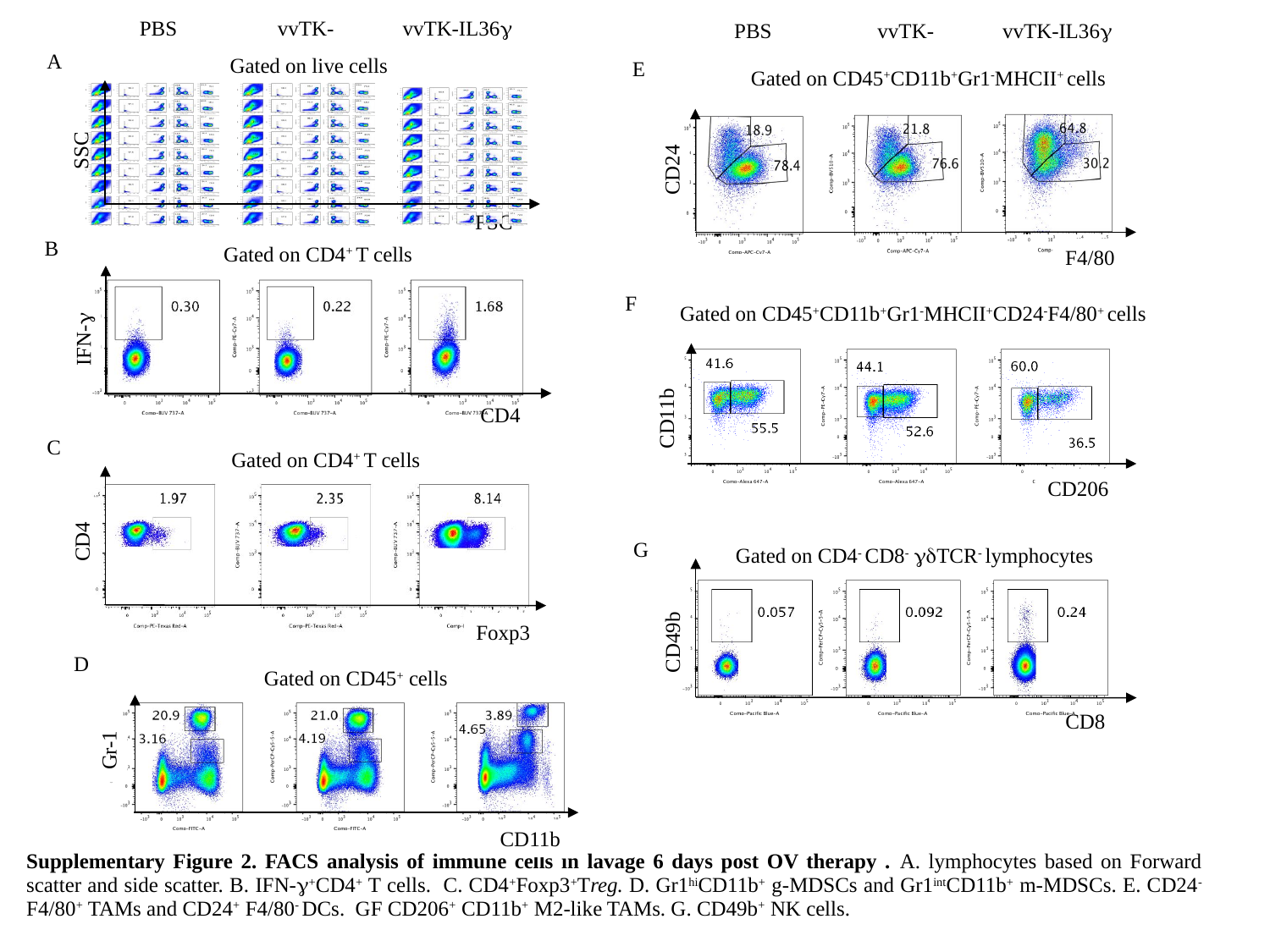

PBS vvTK- vvTK-IL36γ
PBS vvTK- vvTK-IL36γ
A
Gated on live cells
SSC
FSC
E
Gated on CD45+CD11b+Gr1-MHCII+ cells
CD24
F4/80
B
Gated on CD4+ T cells
IFN-γ
CD4
F
Gated on CD45+CD11b+Gr1-MHCII+CD24-F4/80+ cells
CD11b
CD206
C
Gated on CD4+ T cells
CD4
Foxp3
G
Gated on CD4- CD8- TCR- lymphocytes
CD49b
CD8
D
Gated on CD45+ cells
Gr-1
CD11b
# Supplementary Figure 2. FACS analysis of immune cells in lavage 6 days post OV therapy . A. lymphocytes based on Forward scatter and side scatter. B. IFN-γ+CD4+ T cells. C. CD4+Foxp3+Treg. D. Gr1hiCD11b+ g-MDSCs and Gr1intCD11b+ m-MDSCs. E. CD24- F4/80+ TAMs and CD24+ F4/80- DCs. GF CD206+ CD11b+ M2-like TAMs. G. CD49b+ NK cells.

## Slide 3
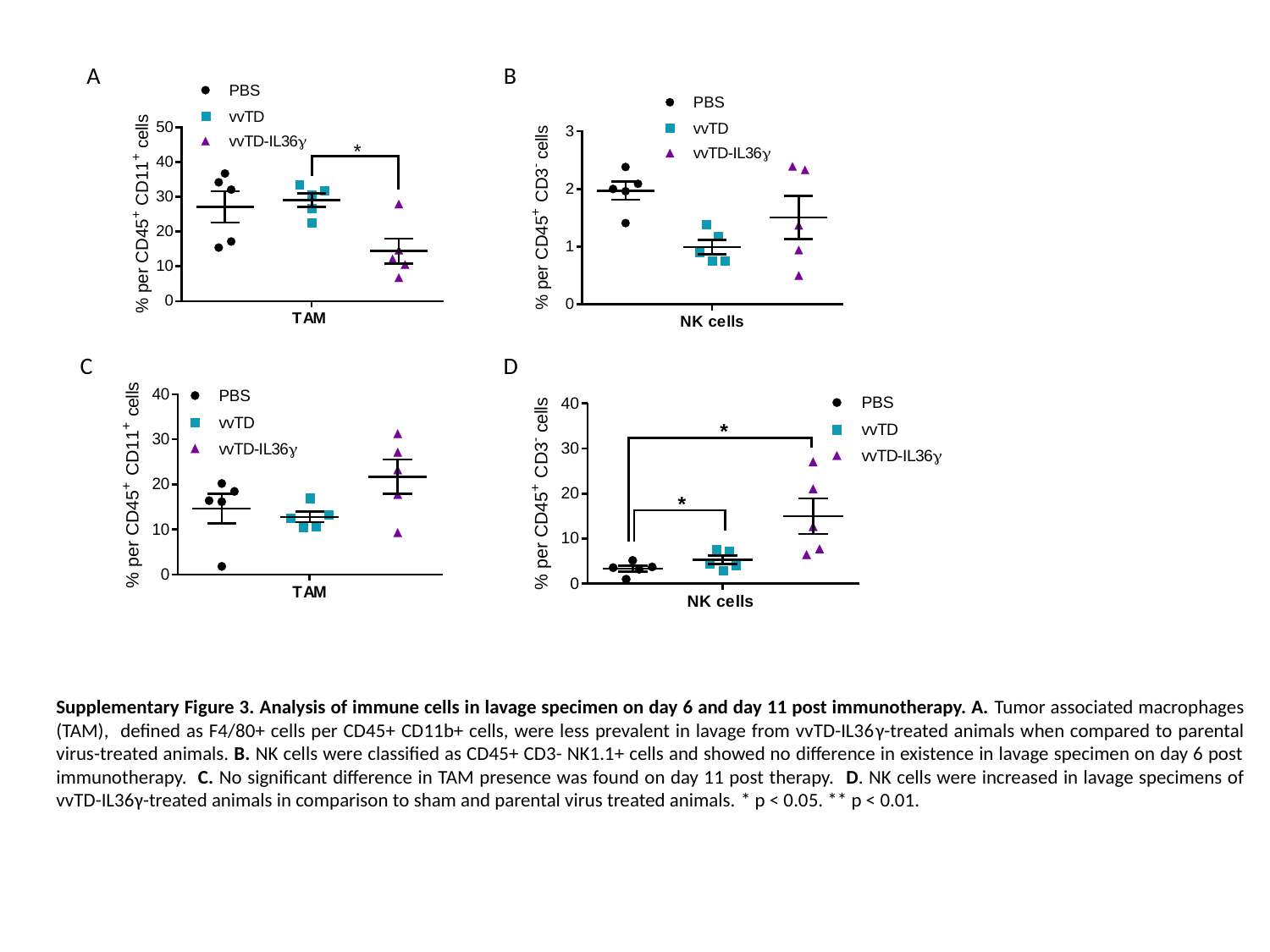

A
B
C
D
Supplementary Figure 3. Analysis of immune cells in lavage specimen on day 6 and day 11 post immunotherapy. A. Tumor associated macrophages (TAM), defined as F4/80+ cells per CD45+ CD11b+ cells, were less prevalent in lavage from vvTD-IL36γ-treated animals when compared to parental virus-treated animals. B. NK cells were classified as CD45+ CD3- NK1.1+ cells and showed no difference in existence in lavage specimen on day 6 post immunotherapy. C. No significant difference in TAM presence was found on day 11 post therapy. D. NK cells were increased in lavage specimens of vvTD-IL36γ-treated animals in comparison to sham and parental virus treated animals. * p < 0.05. ** p < 0.01.

## Slide 4
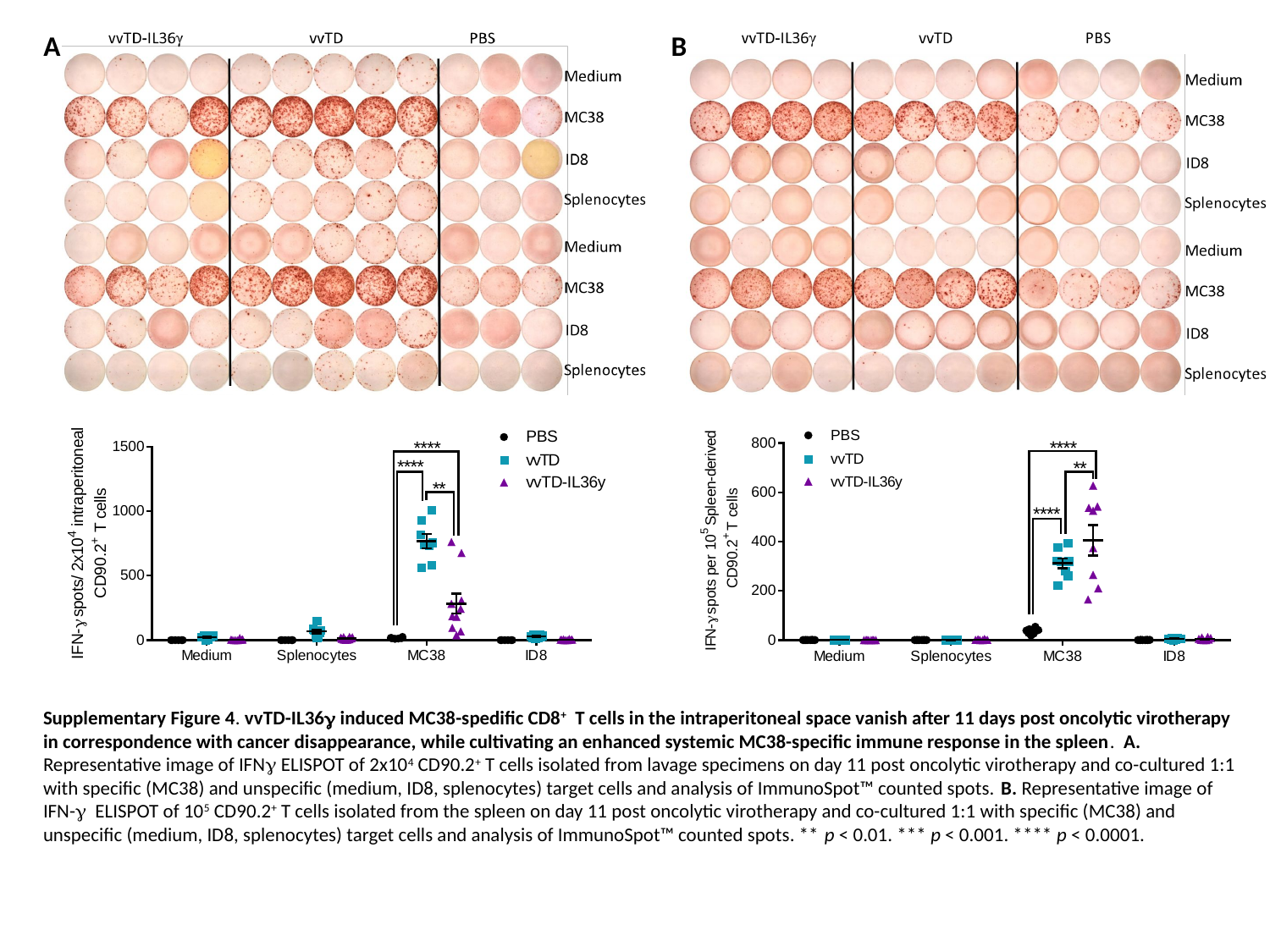

B
A
Supplementary Figure 4. vvTD-IL36γ induced MC38-spedific CD8+ T cells in the intraperitoneal space vanish after 11 days post oncolytic virotherapy in correspondence with cancer disappearance, while cultivating an enhanced systemic MC38-specific immune response in the spleen. A. Representative image of IFNγ ELISPOT of 2x10^4^ CD90.2+ T cells isolated from lavage specimens on day 11 post oncolytic virotherapy and co-cultured 1:1 with specific (MC38) and unspecific (medium, ID8, splenocytes) target cells and analysis of ImmunoSpot™ counted spots. B. Representative image of IFN-γ ELISPOT of 105 CD90.2+ T cells isolated from the spleen on day 11 post oncolytic virotherapy and co-cultured 1:1 with specific (MC38) and unspecific (medium, ID8, splenocytes) target cells and analysis of ImmunoSpot™ counted spots. ** p < 0.01. *** p < 0.001. **** p < 0.0001.
